# ‘FACE ME’—The Impact and Value of an Arts-Based Project About the Patient-Parent-Clinician Relationship in European Reference Network CRANIO

**DOI:** 10.1097/SCS.0000000000011295

**Published:** 2025-03-31

**Authors:** Mariët Faasse, Barbara C. Groot-Sluijsmans, ERN CRANIO FACE ME Project Group, Aafke G. Coopmans, Hester M. van de Bovenkamp

**Affiliations:** * Erasmus School of Health Policy & Management, Erasmus University Rotterdam, Rotterdam; † European Reference Network for rare and/or complex craniofacial anomalies and ear, nose and throat (ENT) disorders (ERN CRANIO), Rotterdam; ‡ Department of Health Science, Amsterdam Public Health, Vrije Universiteit Amsterdam, Amsterdam; § Tranzo, School of Social and Behavioural Sciences, Tilburg University, Tilburg, The Netherlands

**Keywords:** Arts-based methods, boundary objects, craniofacial, ERN CRANIO, professional-patient relations

## Abstract

**Background:**

A good patient-clinician relationship is an important aspect of quality of care according to patients living with a rare congenital craniofacial condition and their parents. Despite efforts, the challenging question remains how to improve this relationship.

**Objective:**

The authors describe the value and impact of using arts-based methods as a catalyst for bringing the 2 ‘worlds’ of patients and their parents and that of clinicians together.

**Methods:**

FACE ME, developed by 2 visual artists, consisted of reorganizing the house of a surgeon with a group of patients (n=3), surgeons (n=3), and a parent (n=1) resulting in 2 artworks. The following data were analyzed using reflexive thematic analysis: the film recording the process, photos of the artworks (i.e., end-results of 2 reorganizations), conversations and reflections of the project group involved, and survey results of respondents who watched the film and photos.

**Results:**

Four intertwined themes describe the impact and value of FACE ME: (1) importance of exchanging trust, (2) seeing each other as a person, (3) imagine standing in each other’s shoes, and 4) working as a team. These themes represent both perspectives and their similarities in expectations of a good relationship, and findings were supported by the views of the project group and the audience watching the film.

**Conclusion:**

FACE ME crossed the boundaries of clinicians, patients, and their parents, and created a better mutual understanding. This arts-based project started a different conversation, and reflection, changed mindsets, and provoked actions towards a better relationship.

People who are born with a rare and complex congenital craniofacial condition often require long-term treatment during their childhood. This consists of frequent hospital visits, and multiple invasive surgeries the number of which depends on the severity of the condition. As physical symptoms (e.g., obstructive sleep apnea, increased intracranial pressure, visual problems, difficulties with eating and drinking) can already occur in early childhood, surgery within the first year is often needed to treat or prevent these problems related to the restricted growth of the skull and/or face.^1–4^ Alongside treatment outcomes, for example, the medical successful outcome of surgery, a good long-term relationship with health care professionals is an essential element of good care according to patients and parents dealing with a craniofacial condition.^5–7^ Studies about the patient-parent-clinician relationship in the area of craniofacial surgery highlight, for example, the importance of a thorough understanding by clinicians of their patient’s individual needs by learning about their experiences, perspectives, and expectations.^5–7^ Furthermore, several systematic reviews in the general literature on the care relationship show that a good patient-clinician relationship and trust are significantly associated with positive health care outcomes. Also, satisfaction with treatment, achieving fundamental patient-centered care, reduced symptoms severity, better health behavior and quality of life over time are related to a good patient-clinician relationship.^8–11^ However, establishing a good relationship proves difficult in practice. This partly has to do with the fact that patients, parents, and clinicians have different ideas about quality of care and life.^12^

To improve the patient-parent-clinician relationship, it is therefore important to better understand each other’s perspectives. One could depict the perspectives of patients, parents and clinicians as different ‘worlds’. In each perspective, the clinician, patient, and parent, brings their knowledge and expectations. On one side there is the world of patients and their parents who carry experiential knowledge, referring to the inner perspective (‘knowing how’, ‘being familiar with’) of patients and parents and what it means to them to cope with the condition in daily life. On the other side, there is the world of clinicians, who bring professional and expert knowledge of individuals with craniofacial conditions.

Individuals experiencing the ‘world’ of the person with the other perspective,^13^ being referred to as boundary crossing, can help to understand each other better. To cross boundaries, arts-based methods are experimented with^14,15^ and artists are increasingly involved. These artists bring performative or representational knowledge, referring to the capacity to visualize what is challenging to express and communicate in words, thereby enabling to transfer lived experiences to others.^16^ By using arts-based methods, artists may develop boundary objects [artwork], evoking emotions among those who created the objects and those encountering these objects.^13^ Such objects can move people from different ‘worlds’ and can create an impulse for change. The more provocative the object, the more people feel triggered to foster change.^13^ Artists can, with these boundary objects, provide novel insights into existing complex issues by creating something that resonates with people, using their curiosity, open-mindedness, imaginative power, and creativity.^16^ They can be seen as boundary spanners as they build bridges between the boundaries of individuals from different perspectives and connect them. By doing so, they play a crucial role in arts-based projects as they act as eminent partners in developing new ways of addressing questions starting from an open position of not-knowing.^14,17^

However, establishing the influence of these arts-based projects on the topic of the patient-parent-clinician relationship remains very limited. Therefore, in this article, we describe the value and impact of using FACE ME, an arts-based project, as a catalyst in bringing the worlds of patients, their parents and of clinicians together by using boundary objects.

## METHODS

### Design

An innovative participatory arts-based project was conducted.^18^ In this project, the goal was, to span boundaries between these different perspectives to be able to improve the patient-parent-clinician relationship. This project took place within the European Reference Network for rare and/or complex craniofacial anomalies and ear, nose, and throat disorders (ERN CRANIO), a network aiming to improve access to and quality of care for these conditions across Europe.

The project started with a question from ERN CRANIO’s coordinator Irene Mathijssen [I.M.]: *“*How can we as health care providers be an even better guide for our patients, by seeing every aspect of their lives, and not just the physical part? And by showing more of ourselves as human beings.” Two arts-health connectors (i.e., spacemakers) [C.H.M., S.B.] who are professionals connecting arts and health care were invited to assist in answering this question. The Creative Catalyst Cycle,^16^ developed by spacemakers and researchers, was used as the approach (Suppl. Table 1, Supplemental Digital Content 1, http://links.lww.com/SCS/H607).

Arts-health connectors [C.H.M., S.B.] invited visual artist-duo [S.Z., H.J.] to participate. The visual artists decided they should live up to their own ideas and personally visit all project participants in their own environment (and due to the distance some virtually) as a patient is more than an individual who needs care, more than just an anomaly. These visual artists used unstructured informal non-recorded conversations online and at people’s homes as their methodology to explore the question. They also visited I.M.’s house. These conversations resulted in the idea to co-create something with the different stakeholders (patients, parents, and clinicians). The project was given the name ‘FACE ME’.

### FACE ME

‘FACE ME’ is about really seeing people and facing them, alongside giving them another face:

“…The point we want to make is the house as a metaphor for the head. When having the surgery you really need to trust your doctor. And we thought we should turn that around. So we asked Irene can we use all your stuff, also the stuff in the cupboard. And she said yes.” [HJ, visual artist]

The visual artist-duo asked if they could use I.M. (plastic surgeon, specialized in the treatment of rare congenital craniofacial conditions) house and everything in it and if the project team including the artists could work there with the whole team for a whole day. I.M. responded positively on this request and a day was scheduled. Specifically, the visual artist-duo invited patients (n=3), surgeons (n=3), and a parent (n=1), and asked them to change I.M.’s house completely and guided the group to make 2 different pieces of artwork with her possessions. A list of invited guests, from now on referred to as project members and their roles can be found in the Suppl. Table 2, Supplemental Digital Content 1, http://links.lww.com/SCS/H608. This led to 2 artworks that they could photograph. Cameras were placed in the entire house to audio- and video-record this process. The video material was used to develop the film FACE ME. While making the artworks, artists gave some small hints on purpose (e.g., to block the light coming in through the bathroom window) to reach the goal of the artwork even though the project team did experience most happenings as a coincidence.

**FIGURE 1:**
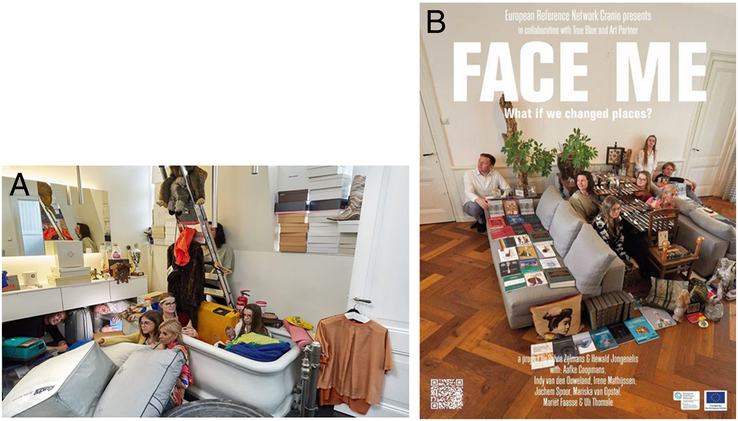
Posters designed by the visual artists. (A) Result of the first session. (B) Result of the second session.

After this, FACE ME continued. The short film^19^ and 2 photos (Fig. 1) produced, were used for a wider audience of patients, parents, and clinicians in workshops designed by the visual artists [C.H.M., S.B.]. Also, an audience of clinicians and patient representatives viewed the film at ERN CRANIO’s Annual Meeting in Dublin. In all instances, the FACE ME film was used as the starting point to open a dialog between patients, parents, and clinicians about the patient-parent-clinician relationship.

### Data collection

A qualitative multi-methods approach was used to gain an in-depth understanding of the value and impact of FACE ME related to the patient-parent-clinician relationship. First, the experiences, observations, and reflections of the artists and members of the project group were collected with prior consent by notes from individual online conversations with the project members, and public reflections (quotes) of some members were recorded during the film presentation. Furthermore, the short film, and the reactions of the audience to the film and participants of the workshops were collected by an online open-ended question survey (Suppl. Table 1, Supplemental Digital Content 1, http://links.lww.com/SCS/H607).

### Analysis

A reflexive thematic analysis,^20^ as an inductive iterative approach, was conducted to study the influence of the arts-based project on boundary crossing between patients, parents, and clinicians. In the first part, (development of film and artwork in the house) FACE ME served as a boundary object for the project team members who were involved. In the second part (film & photos), FACE ME can be considered as a boundary object for the *audience* (e.g., patients, parents, clinicians) watching the film during the presentation and workshops. For both parts, boundary crossing was analyzed and described separately.

The first author [M.F.], a patient representative, researcher with a clinical background, and participant in the FACE ME project, carried out the initial analysis. This started with the analysis of the survey responses. Firstly, the first author became familiar with the data after reading it multiple times. Secondly, responses were coded based on the meaning of its content. Finally, codes were put together in groups describing different themes in the data. Themes and findings were then discussed thoroughly among the authors who are a mixture of interviewed clinicians, participants in the film, visual artists, project managers involved in FACE ME, and researchers with expertise in arts-based research.

As for the analysis of survey data, also the notes of the informal conversations with all the project members in the film, and the film itself were, respectively, read and watched several times by the first author. Video stills supporting identified themes in the notes of the conversations with project group members were described narratively. The analysis was discussed with the authors in online and individual meetings multiple times and adapted.

## RESULTS

The FACE ME project served as a boundary object, which can be described by the following 4 themes: exchanging trust, seeing each other as a person, imagining standing in each other’s’ shoes, and working as a team. The themes, all illustrated by a video still, are described below in the we-perspective, referring to the project members involved in the project (see Suppl. Table 2, Supplemental Digital Content 2, http://links.lww.com/SCS/H608). The quotes in the text illustrate the views of a clinician (C) or patient/parent (P).

### The Importance of Exchanging Trust

FACE ME made the importance of trust tangible in different ways, emphasizing that establishing trust requires work from both sides: the patients and their parents and the clinician.

#### Trust—While Reorganizing the House

**FIGURE 2:**
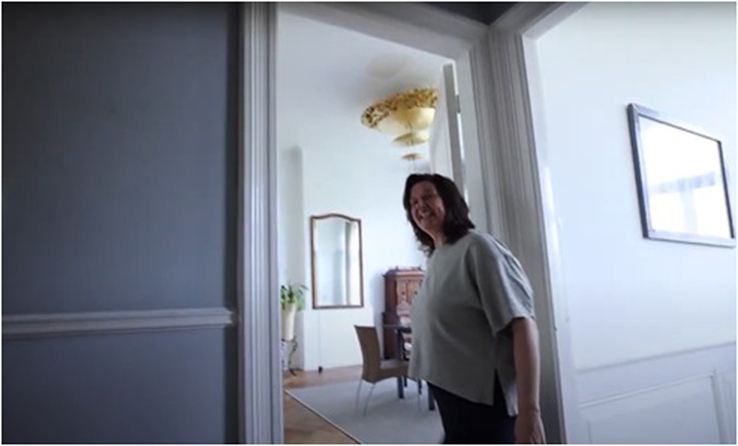
Video still [1.30 min] “Just follow me”—(I.M., plastic surgeon, house owner) is giving a tour of her house, inviting us to come in.

We were invited to open every drawer, closet, box, and door of the entire house. By giving access to all her personal belongings I.M. provided insight into her personal space and private life (Fig. 2). At the start, we, apart from I.M. [owner house] were hesitant to execute the project as explained: without rules, without roles. We were in doubt what was appropriate in the space between us guests and I.M. in our specific roles and relationships with her. This created the feeling that it was not up to us to change the house as you may not even do this with your friends or family. Once we passed the front door, we felt we crossed a privacy threshold. After several conversations, we were encouraged by the owner of the house and the visual artists that every object was allowed to be moved. Puzzling thoughts stayed in our minds: Why are we allowed to do this? ‘Was it right to do this?’… ‘How do you feel you might have crossed a (privacy) line for the IM (plastic surgeon)?’ What if something is broken? What can we touch and take without creating discomfort or emotionally hurting the owner? This situation of ‘giving up control’ was her giving trust back to her patients and their parents. We learned that by allowing people to reorganize your house, or by analogy, allowing invasive surgery on your face, there always needs to be a legitimate reason and necessity for accepting that. In other words, to submit your child or yourself to such a treatment only feels acceptable when you know there is no other option left. In the hospital, patients and parents are not in the position of choosing to let clinicians into their lives, rather it happens to them as a necessary consequence. With this in mind, we as a group experienced this day as a gift, understanding what it costs to give up a part of your privacy and control whether involuntarily or not.

#### Trust—While Watching the Film

As a parent after the workshop stated: “For something as drastic as facial / skull surgery, a lot of confidence is needed in the other (the physician). This confidence can be increased if the physician really has an eye for the interests and perceptions of the patient. ” (P) Also, watching the short film created an awareness of the importance of trust leading to further reflections and conversations during the workshops. It shows how the FACE ME project incites reflection on the patient-parent-clinician relationship by the patients while stating the influence of their own contributions:

“Yes, the doctors hear what patients / parents are running into, or what they need, or what is helpful in a good relationship so that there is mutual trust. If you as a parent/patient always approach the doctor with mistrust, it is just as annoying as the other way around. If both parties open up to each other, trust can arise.” (P)

Clinicians expressed they expect patients and their parents to be open and honest about their thoughts, expectations, and experiences, for which trust is needed. In doing so, it makes it easier for clinicians to be open when discussing concerns, perform optimally, and be a better guide during the treatment pathway. In line with these experiences, clinicians themselves also concluded that the trust of patients and parents in them is crucial. Meanwhile, clinicians reflected on the impact of the treatment they provide on their patients:

“Understanding how patients trustfully put their lives and bodies in the hand of a surgeon. We [clinicians] need to have this clear in mind to improve the empathy in interactions with them.” (C)

Elements that, according to participants, facilitate trust in clinicians were: being open as a person, being honest about treatment plans and prognosis, showing empathy, having space to express feelings, and involving the patient when being a child in the conversation. Furthermore, they discussed that a clinician’s understanding, respect, giving control back as much as possible, providing information, and using shared-decision-making were crucial elements for trust. However, they recognized the challenge of putting this effectively into practice and discussed the hurdles they encountered personally. This, on its own, resulted in a better mutual understanding of each other’s perspectives.

### Seeing Each Other as a Person

FACE ME illustrated the complexity of balancing desired proximity and keeping a safe distance, which resulted in seeing each other as a person.

#### Everyone as a Person—While Reorganizing the House

**FIGURE 3:**
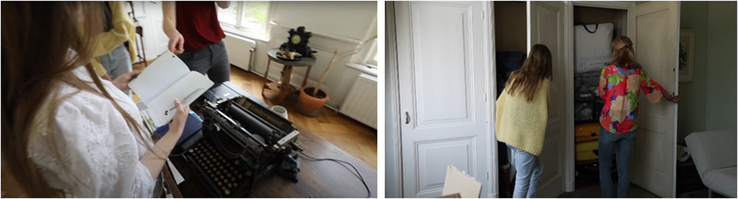
[3.25 min] “Look this is the diary of I.M. (house owner). We can open everything, right?” A.C. [patient], I.O. [patient], and M.F. [patient] (photo 1) opening a dairy/notebook and I.O. [patient] and M.O. [parent] (photo 2) the wardrobes.

When we were rearranging objects in different rooms, it felt very much like we were diving into the owner’s [I.M.] personal space (Fig. 3). In a regular conversation we can always decide how much we want to show of ourselves, although in this situation, there were no boundaries. It also showed us the person behind the surgeon, to see what I.M. likes and finds important in her personal life outside the hospital. This situation made us realize that we always only see a part of someone when being the patient, doctor, or colleague of the other person even when we have an eye for each other’s personal life.

Working and being with persons you only know from the hospital environment also brought back memories from that place. However, because we were not in the clinical setting, we noticed it became easier to put aside these positive and negative hospital memories. In this way, it became easier to see each other without these connected experiences as they disappeared into the background. Not only because of the environment but also because in this setting we had time and freedom, which created the feeling of a more open atmosphere to see each other as a person.

Being a surgeon takes more than ‘skills in the operating room’, and a patient is more than a condition or diagnosis. Similarly, objects in a house often carry several stories, just as patients and their families have their stories, experiences, and thoughts. For us, this raised multiple questions:

‘How much (personal) information did we get being in IM’s house that we were not supposed to get?’ [JS, neurosurgeon].

Was there a locked drawer or door with off-limit stuff behind it? Similarly, when being in hospital, patients’ stories or experiences are both explicitly and unintentionally shared. Although we were all busy in the house, some small elements in the 2 artworks (i.e., the 2 photos) still referred to our positions and experiences in the real world some, but not all, of them being shared with the group. There was the image of looking at the back of the mirror turning away from yourself, but confronting the audience to see themselves instead (A.C., patient, Fig. 1A), a tape measure taken from a sewing box around a head as a memory for the head circumference measurement in hospital (M.F., patient, Fig. 1A), and silverware placed on the tables and organized like surgical instruments (all, Fig. 1B). Furthermore, it happened that I.M. started telling stories about some of the objects we encountered. This situation further blurred boundaries between the project members by stressing how similar we actually are as human beings. The experience of getting to know someone’s personal life versus giving enough privacy and space created tension. It is all about finding the right balance between showing personal interest and making a connection versus being too curious and coming uncomfortably close.

#### Everyone as a Person—While Watching the Film

Participants in the workshop and those who watched the film express the need for some encouragement to show and be yourself in hospital as a patient or parent:

“Name the power imbalance and do not be afraid to show honest feelings and vulnerabilities.” (C)

In contrast, when you get to see more of a person it can make you more open toward that person:

“See one behind the condition, see me in more facets than just the medical problem I present now.” (P)

Or to see the impact of treatments taking place in hospital:

“Look what you are doing to me” (P)

Participants in the workshop started reflecting on their relationship as a connection between humans and a feeling of: “being with” (C) and “take time to find out from the patient what they need help with instead of assuming you know their needs” (C). This led to some concrete ideas of actions to work towards this goal:

“Good patient information: pictures, personal predictions, practical information, would save time and leave room for a more meaningful and understanding conversation.” (C)

As a result of becoming aware of the importance of seeing each other as a person, the focus and intentions of conversations between patients, parents, and clinicians changed.

### Imagine Standing in Each Other’s Shoes

FACE ME made it possible for people to take the other’s perspective, which subsequently contributed to respect for and a better understanding and of each other.

#### In Each Other’s Shoes—While Reorganizing the House

The day started very open without detailed instructions. This left us as a group in uncertainty about how to behave and what to do. UT (neurosurgeon) shared his thoughts about this moment at the premiere of the film. He illustrates the predictability and clarity of situations he is used to while working in his hospital and the contrast with this project:

“… and actually of course there was some kind of idea that we should switch our roles. But at the end we normally have in our hospitals like the written consent, we have diagnostics done, we talk about risks and complications and so on. That was not there and most peculiar: there was no purpose. No obvious purpose at least. So these were the things which were completely different…

This uncertainty and unclarity, which are often very familiar feelings to patients and parents in the hospital, was now also felt by the surgeons involved.

**FIGURE 4:**
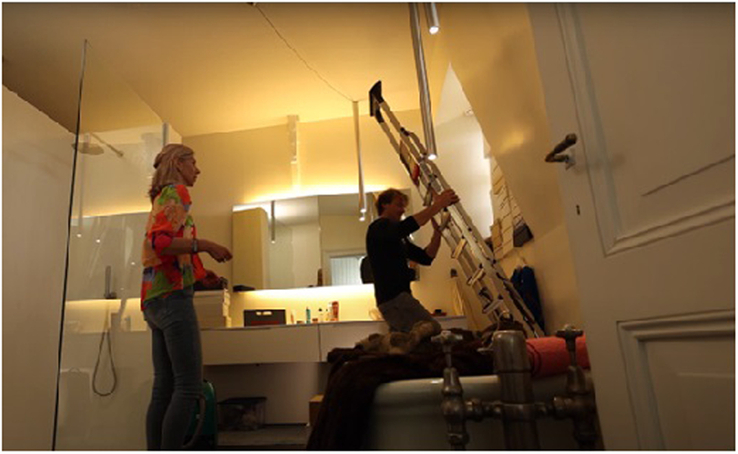
Video still [5.12 min]*“*Are you not afraid of damaging something?” [M.O., mother] “Yes, quite a bit.” [J.S.] Neurosurgeon [J.S.], holding the ladder very carefully placing it behind the bath.

During the project, it stayed in our minds that we had to be careful and wanted to treat I.M.’s belongings with respect and, therefore, had ongoing concerns about damaging objects that are of value to her. In the living room, we (the guests) all put on gloves wanting to be very careful and show respect. Equally, patients and their parents are at the mercy of their clinician, which can feel very vulnerable, both with regards to undergoing treatment and living with a craniofacial condition. Consequently, clinicians feel a high responsibility to do good and not (further) harm when treating a patient. As an example, the “ladder-shot” (Fig. 4), was associated by a lot of people with feelings and experiences of risk-associated surgery on a (little) patient in hospital. It expresses and envisions the feeling of invasiveness and associated risks and, at the same time, the accuracy of how clinicians handle these.

#### In Each Other’s Shoes—While Watching the Film

The film created a feeling of changing places in an analogy that made it easier to empathize with the other person:

“It [FACE ME film] lets you feel the loss of control that patients have when they have to trust you for their treatment.” (C)

Several survey respondents stated that seeing the film makes clinicians ‘reflect on the position they put their patients in’. They also alluded to the strength of the FACE ME film as it is “hard to get out of your own shoes” (C) in daily life and the clinical setting. Some of the participants watching the film expressed the potential of FACE ME being used in other settings and how this could help patients to be better understood and get prepared for treatments:

“I am very curious about how the project will proceed. I hope that something will also come out for patients themselves, so that they can give more words to what it means for them to undergo surgeries/procedures.”(P)

The film initiated a conversation allowing people to be better understood in their experiences and giving words to their feelings.

### Working as a Team

FACE ME showed that working together enhanced a better bond with time to get to know each other and each other’s contributions as important aspects.

#### A Team—While Reorganizing the House

**FIGURE 5:**
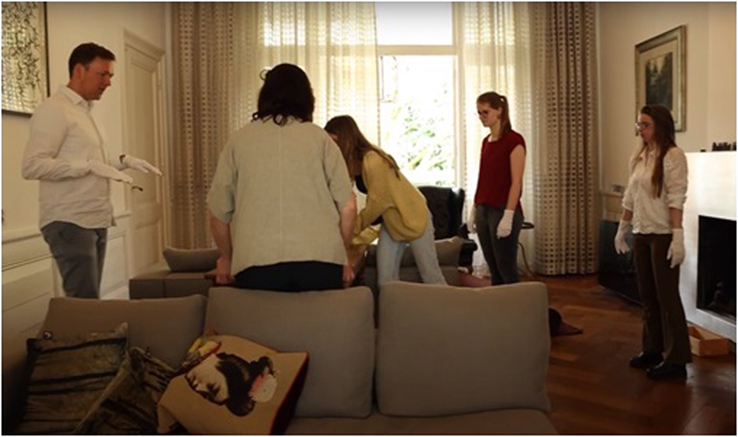
Video still [8.08 min] “I am happy with you all.” [I.M., plastic surgeon, owner house] “We can try to put as many tables and then it goes… like stairs down.” [U.T., neurosurgeon] “Okay.” [I.M.].

Although we were redesigning the house we felt equal to each other in our contributions to the work in progress. Initially, we started as individuals in the first part (Fig. 2A: the bathroom) which therefore was more random and chaotic. After a while, when we felt more at home, in the second part (Figs. 5, 2B: the living room) we made arrangements, had a plan, and started working together more resulting in a very structured and attuned performance:

“Our role in regular life disappeared, instead a powerful team spirit developed naturally.” [UT, neurosurgeon].

In contrast to our natural roles when being in the hospital this setting was more about collaboration rather than information exchange. We could be ourselves: it was without roles, labels, hierarchy, or being surrounded by doctors ‘looking at you’ to see what needs to be done. This enabled us to act beyond the boundaries of our traditional roles. When reflecting, the certain level of hierarchy there is between patients, their parents, and clinicians is traditionally hard-wired and also influences the ways we naturally behave in these roles.

#### A Team—While Watching the Film

Workshop participants and people who viewed the short film experienced similar feelings. It created an open dialog and atmosphere in which patients, parents, and clinicians felt comfortable to express their thoughts without hesitation:

“This was not a clinical setting. The project was not at a time of making choices about treatments. It was very accessible. I really enjoyed talking to my son’s health care professionals in this way. Welcoming, like equals.” (P)

Furthermore, the film created a more approachable atmosphere: “The project could be a way to start the conversation, to lower thresholds.” (C). Respondents commented about a need for change and desire for a “paradigm shift” to “making a real shared-decision” (P), and a wake-up call to “educate doctors on the reasons why change must happen” (P) to work together more:

“I truly enjoyed it but it also moved me emotionally. I want to see patients and clinicians as people interacting.” (C)

The ultimate result was a feeling of togetherness without boundaries to discuss all thoughts that came to mind.

## DISCUSSION

This study aimed to gain new insights into the traditional patient-parent-clinician relationship and to start a different conversation between these individuals through the art-based project FACE ME. During this project, the art development process, the film, and photos served as boundary objects. This became apparent in the narrative description of several cognitive conflicts, contradictions, thoughts, feelings, and experiences FACE ME revealed for clinicians, parents, and patients. By the use of boundary objects in this project, boundary crossing could take place. This ultimately resulted in boundary spanning in terms of patients, parents, and clinicians connecting and bridging each other’s perspectives and ‘worlds’.

We identified the importance of exchanging trust, seeing each other as a person, standing in each other’s shoes and working as a team as key elements in Face Me. Interestingly, we found that themes were intertwined in which the importance of exchanging trust, seeing each other as a person were elements that would follow imagining standing in each other’s shoes. Furthermore, time and effort are needed to be able to work as a team. These elements shed light on the patient-parent-clinician relationship and shows the value and impact of FACE ME. The next sections describe the scientific and practical lessons of FACE ME in more depth.

### From Co-Creation to Relation in Action

Our findings show that for the audience and the project team FACE ME felt like an exercise of building a relationship ‘in action’. While exploring the starting question, we did not only find an answer, but the answer immediately became a real practice in this project. Subsequently, as far as we know, our project is the first one in the craniofacial field to approach the patient-parent-clinician relationship as a collaboration and co-creation rather than the emphasis on the responsibility of clinicians only to make the relationship work.^21^ Even though the project started from the perspective of a clinician to come closer and be a better guide for clinicians, patients, and their parents interestingly could also step in the other’s shoes and see through the eyes of the clinicians. Other tools to exchange experiences and information have been developed, such as mirror meetings.^22^ These often place the 2 different parties (i.e., patients and clinicians) in front of or behind each other. In FACE ME; however, we brought patients (and parents) and clinicians together in their relationship getting a better understanding of each other’s experiences. In other words, our approach was a collaboration to better understand and learn from experiences rather than a transaction of experiences and information exchange. Similar approaches are experience-based co-designs that combine collaboration between patients and clinicians with arts-based elements such as videos to work towards quality improvement.^23,24^

### FACE ME

The visual artists created the opportunity to experience the other perspective. They changed traditional places of patients, parents, and clinicians by stepping in to each other’s world. Specifically, the house and everything in it as a metaphor and familiar object, truly standing in the middle of worlds of patients, parents, and clinicians created a tangible situation that all, in their personal lives, could relate to. The focus of the patient-clinician relationship in research and enhancement in clinical practices has mainly been on the cognitive part (i.e., information gathering, sharing medical information, patient education, and expectation management)^8^ by holding webinars, developing information leaflets, and giving patients and parents access to electronic medical records. Conversely, the emotional part (i.e., mutual trust, empathy, respect, genuineness, acceptance, warmth),^8,11^ receives less attention, probably due to its difficulty in operationalization and measurement of these interpersonal elements in clinical practice and research.^8^ In FACE ME and this paper, we tried to uncover these elements specifically to give clinicians as well as patients and parents better insights and to provide some tools to work on these aspects. As a result of a better understanding, our study also adds a better description of the sense, meaning, and impact these elements might have on the literature.

### Value of Arts as Boundary Object

Previous literature states the complexity of the patient-clinician relationship and therefore this interdisciplinary approach of using arts may be an appropriate response addressing and exploring this topic from a different standpoint.^13,25^ Using visual artists with an open and different view in our project led to boundary objects that could be used to create boundary spanning in different ways. The boundary spanners, which led to actions and interpersonal changes, could not have been identified without using visual artists or other approaches to study the patient-parent-clinician relationship. In addition, rather than describing the patient-parent-clinician relationship only, it allowed individuals to express their experiences and feelings in an open atmosphere, which are often difficult to understand and communicate.^26^ Arts-based research and approaches share theoretical concepts, such as boundary objects, with the fields of sociology, anthropology, philosophy, and psychology and use a qualitative, social sciences approach to determine the outcomes and impact of projects.^25^ Therefore, evaluating FACE ME’s impact using a biomedical model, the premise for health care, that values scientific, quantitative data, and experimental approaches is challenging and does not do justice to this project. As authors from different disciplines, working with the complexity of this interdisciplinary field, we described our analysis as mainly exploratory rather than explanatory. Through photos and stories, we have tried to describe the project and explain some possible theoretical mechanisms of transformative participatory arts-based projects in health care settings.

### Clinical Implications

Workshop participants and the audience who viewed the film highlighted the power of FACE ME as a reflection and learning tool. Because of the interest of several clinicians and patient representatives in ERN CRANIO to deliver the workshops with FACE ME to their local teams and organizations an online train-the-trainer session has been held. For other patients, parents, and clinicians, the FACE ME film could be a valuable intervention tool to start a(n) group-based) conversation about things difficult to express in words to strengthen relationships. Furthermore, as a reflective tool, the film could be used to give patients and their parents better words to what they or others might experience. Lastly, the film could be used as an educational tool and information source to learn what is important to patients, parents, and clinicians, and if necessary local practices or their own approaches can be adapted accordingly.

### Strengths and Limitations

For analysis, we used multiple qualitative sources (i.e. observations of the film, notes from conversations of project members, and survey results). This data triangulation^27^ enabled us to give a more detailed, balanced, robust, and complete description of the value and impact of FACE ME. As the conversations with the project members are not recorded, verbatim transcripts could not be provided, thereby limiting the transparency of data analysis. However, conversations were extensively described during the conversations and we repeatedly reflected together on the project and the writing of this paper. As the arts do not have the purpose to be explained and understood in one single way, insights and interpretations of the authors and survey respondents are not exclusive and other views can simultaneously be true. Also, other contexts might have a different effect on the outcomes of such a project. In this paper, we describe 2 ways in which we offered FACE ME to the project members and the audience and participants, respectively. However, there can be more ways to present FACE ME that might be relevant and valuable. Of importance is to note that we did not take into account the differences in perspectives between patients and their parents in this project and our analysis. As we know from previous literature^7^ that these are not necessarily the same, this can be seen as a limitation to our study.

### Recommendations for Future Investigations

Throughout the paper, we were using the term patient-parent-clinician relationship as in the care of congenital craniofacial conditions parents, or caregivers, play a very important role. However, we did not look specifically at the interactions between (young) patients and their parents, their difference in perspectives or the role of family for both patients and clinicians in this relationship. To investigate this, the visual artists and arts-health connectors completed a follow-up project: ‘WITH MY FAMILY TO THE THEATRE’^28^ in Paris, France about the role of family. It shows the significant role of family, but also how their constant presence can sometimes be uncomfortable for both doctor, patient, and family members and how this might influence the persons in this relationship. Secondly, there is an urgent need to further explore the potential of arts-based projects in this complex multidisciplinary medical field as current data is scarce and thereby evidence limited. Finally, we need to look at how we can implement and integrate the lessons learned from these arts-based interventions into clinical practice.

In conclusion, FACE ME offered a unique and new way of looking at the multi-level patient-parent-clinician relationship, emphasizing the mutuality in this relationship, and inspiring people to cross the boundaries of perspectives resulting in a better mutual understanding.

## Supplementary Material

SUPPLEMENTARY MATERIAL

## ERN CRANIO FACE ME PROJECT GROUP COLLABORATORS

Sandra Boer MA, Art-Partner, Amsterdam, The Netherlands, Cathy Hassels Mönning MA, True Blue - Creative Healthcare Communications, Amsterdam, Netherlands, Hewald W.J.M. Jongenelis, Zijlmans & Jongenelis, Amsterdam, The Netherlands, M. Kljajic PhD, The Sahlgrenska Academy, Institute of Clinical Sciences, University of Gothenburg, Department of Plastic Surgery, Sahlgrenska University Hospital, Gothenburg, Sweden, Irene M.J. Mathijssen MD PhD, Department of Plastic and Reconstructive Surgery, Erasmus University Medical Centre Rotterdam, Rotterdam, The Netherlands; European Reference Network for rare and/or complex craniofacial anomalies and ear, nose and throat (ENT) disorders (ERN CRANIO), Rotterdam, The Netherlands, Mariska van Opstal, Project member FACE ME, Indy van den Ouweland, Project member FACE ME, Jochem K.H. Spoor MD PhD LLM, Department of Neurosurgery, Erasmus MCSophia Children's Hospital, Rotterdam, The Netherlands, Ulrich-Wilhelm Thomale MD PhD, Pediatric Neurosurgery, Charité-Universitätsmedizin Berlin, Corporate Member of Freie Universität Berlin and Humboldt-Universität zu Berlin, Berlin, Germany, Sylvie Zijlmans, Zijlmans & Jongenelis, Amsterdam, The Netherlands.
