# Supplementary material for: ‘FACE ME’—The Impact and Value of an Arts-Based Project About the Patient-Parent-Clinician Relationship in European Reference Network CRANIO
Source: J Craniofac Surg. 2025 Mar 31;36(8):3004–12. doi: 10.1097/SCS.0000000000011295 (PMC12537040; doi:10.1097/SCS.0000000000011295)
Supplement: SUPPLEMENTARY MATERIAL [file scs-36-03004-s001.docx]

| Table 1. Development of the FACE ME project [19] using the Creative Catalyst Cycle [16]. | | | | |
| --- | --- | --- | --- | --- |
| Phases | **Description** | **Application FACE ME project** | **People involved** | **Data output** |
| Need for change | Defining the wish or question with the customer: Where and what is the desire (or pain) in the organisation? | Relationship between patient, parent and clinician. | Initiator of the project & two art-to-health connectors (spacemakers) |  |
| Uncover | Through qualitative artistic research with artists it will be uncovered what’s going on. Dynamics, patterns and emotions are brought to the surface via various methods. | Informal unstructured conversations (at home, online, at work) | Clinicians from different countries and patients and their parents conducted by two visual artists and two arts-to-health connectors (spacemakers). | Written notes of artists. |
| Envision | What information has been collected? Together with the costumer a shared ambition will be determined. Insights, needs and ideas will be put together to specify the wish/need for change and the shared experienced vision of what is needed. | Re-defined question: *"How can we as doctors and healthcare providers be an even better guide for our patients, by seeing every aspect of their lives, and not just the physical part? And by showing more of ourselves as human beings."* | Initiator of the project and project manager with visual artists and arts-to-health connectors (spacemakers). | Written notes of artists. |
| In motion | Information with be used to develop an artistic intervention tailored to the organisation and question. Proven art techniques from theatre, music and visual art can be used. During this phase stakeholders will feel and experience what is going on and this triggers the start of the first motion. | The co-creation of ‘FACE ME’: a video and two photos.    FACE ME sessions:  1. Workshop try-out with clinicians craniofacial team and adults living with and parents of children with craniofacial conditions from patient organisations  2. Workshop at Annual Meeting ERN CRANIO, Dublin  3. Plenary premier presentation FACE ME project (film and photos) at ERN CRANIO Annual Meeting  4. Online train-the-trainer session for the workshop as response to the desires of clinicians in the network to lead workshops locally. | *FACE ME video:* surgeons (n=3), patients with a craniofacial condition (n=3) and a parent supported by two visual artists.  *FACE ME workshop NL & IE:* clinicians of craniofacial teams (n=14) and adults/parents/pt representatives living/dealing with various craniofacial conditions (n=16).  *Plenary presentation IE:* clinicians and patient representatives (n=139) | FACE ME video + photos, audio recordings + notes from reflexive conversations with project members. |
| Harvest | What is needed to increase the impact and to keep the motion going? The harvest of the trajectory will be brought back to the organisation and embedded in the organisation: daily practice and life. Tailored tools can be developed connected to the art intervention (e.g. workshops, trainings, education programs) in which experience-based learning and other reflection and interaction standards are used. | Reflections of participants of project/workshop, the interest of other medical specialties in the project, desire to continue workshops. | People directly involved in phase 4 (in motion), and their connections/contacts in hospitals, disciplines patient communities. | Input from the survey of participants at workshops, presentation plenary session (n=33) |
| Reflect & Continue | What did it bring, what is missing, what did (not) work? This will guide the future steps. | Reflections on the project, e.g. resulting in a new film “With my family to the theatre” focussing on the role of families of patients and clinicians. | Authors of this article, ERN CRANIO network, patient(s) (organisations), clinicians. | See discussion. |
