# Supplementary material for: ‘FACE ME’—The Impact and Value of an Arts-Based Project About the Patient-Parent-Clinician Relationship in European Reference Network CRANIO
Source: J Craniofac Surg. 2025 Mar 31;36(8):3004–12. doi: 10.1097/SCS.0000000000011295 (PMC12537040; doi:10.1097/SCS.0000000000011295)
Supplement: SUPPLEMENTARY MATERIAL [file scs-36-03004-s002.docx]

| **Table 2:** People involved in creating the short film in the house. | | | |
| --- | --- | --- | --- |
|  | **Name** | **Role** | **Extra info** |
| Surgeons | Irene Mathijssen [IM] | Plastic surgeon | Owner of the house in the film |
|  | Jochem Spoor [JS] | Neurosurgeon | Direct colleague of IM |
|  | Ulrich-Wilhelm Thomale [UT] | Neurosurgeon | Colleague of IM and JS from Germany |
| Patients with a craniofacial condition (n=3) | Aafke Coopmans | Patient | Patient |
|  | Mariët Faasse | Patient | Patient (representative) |
|  | Indy van de Ouwehand [IO] | Patient | Patient |
| Family member of a patient | Mariska van Opstal [MO] | Parent | Mother |
| Visual artists | Hewald Jongenelis [HJ] & Sylvie Zijlmans [SZ] | Guiding the group |  |
